# *IN VITRO* AND *IN SILICO* ANTIBACTERIAL ACTIVITIES OF *SYZGIUM AROMATICUM* ESSENTIAL OIL AGAINST BACTERIA ASSOCIATED WITH *OTITIS MEDIA* IN CHILDREN

**DOI:** 10.21010/Ajidv17i1.3

**Published:** 2022-12-22

**Authors:** OBUOTOR Tolulope Mobolaji, ADEYANJU Folasade Oluwademilade, KOLAWOLE Amos Oyebisi, IDOWU Gbohunmi Paul, OLUWAMUYIWA Fesobi Andrew, AFOLABI Felix Olaide

**Affiliations:** 1 Department of Microbiology, Federal University of Agriculture Abeokuta, Ogun State, Nigeria; 2 Department of Biochemistry, Benjamin Carson College of Medicine, Babcock University, Remo, Ogun State, Nigeria; 3 Department of Microbiology, Covenant University, Ota Ogun state, Nigeria; 4 Department of Pharmacology, Ondo State University of Medical Sciences, Ondo, Ondo State, Nigeria

**Keywords:** *Otitis media*, Clove, Antibacterial, Essential oil, MIC, *In silico*

## Abstract

**Background:**

This study investigated the efficacy of the essential oil (EO) of *Syzgium aromaticum L.* (clove) on the bacteria associated with *otitis media*.

**Materials and Methods:**

Ear swab samples were collected and bacteria isolated were identified using morphological and biochemical procedures. Essential oil was extracted from the dried flower buds using the hydro-distillation method while physicochemical and phytochemical analysis was done on the oil. Antibiotic susceptibility test and agar well diffusion was used to determine the susceptibility of the isolates to the EO. In – silico analysis was conducted to determine the drugable compound in the EO.

**Results:**

Phytochemical analysis of the oil indicated the presence of flavonoids, sterols, phenols, carbohydrates and alkaloids. Physicochemical test of the EO exhibited the presence of eugenol (80.98%) as the most abundant phytocompound. Percentage occurrence of the bacterial isolates are as follows; *Proteus mirabilis* (31.25%), *Staphylococcus aureus* (25%), *Pseudomonas aeruginosa* (18.25%), *Proteus vulgaris* (3.12%), *Moraxella catarrhalis* (12.5%), *Klebsiella pneumoniae* (3.12%) and *Staphylococcus epidermidis* (6.25%). Zones of inhibition were within the range of 11.5±0.71mm-23.0±2.83mm. In silico studies demonstrated that 16 out of 38 compounds identified passed the ADMET analysis. Various compounds had different binding energies, such as linalool, carvacrol for *S. aureus* (2NOJ), gamma-eudesmol, eudesmol for *Proteus mirabilis* (6H2L), eucalyptol, gamma-eudesmol and eudesmol for *Proteus vulgaris* (4MCX) and *Staphylococcus epidermidis* (4HBL).

**Conclusion:**

This study shows the potency of clove EO as an antibacterial agent and its component as potential lead molecules in drug development and design to combat multi – drug resistance.

## Introduction

*Otitis media* (OM) refers to a group of complex infectious and inflammatory diseases affecting the middle ear (Dickson, 2014). This occurs when the middle ear drum and the inner ear, including the Eustachian tube becomes inflamed (Arroll, 2005) and it is a common problem worldwide (Cripps and Kyd, 2003). Egbe *et al.*, (2010) reported that children less than 5 years are more susceptible to otitis media infection and this is due to their shorter and more horizontal Eustachian tube. Children also have lower immunity compared to adults and bacteria tend to attach to the epithelial cells of children than in adults. OM may be presented in different clinical forms including “Acute otitis media (AOM)”, “Otitis media with effusion (OME)” and “Chronic suppurative otitis media (CSOM)”. Sierra *et al.*, 2011 opined that the most common cause of OM is the bacterial infection of the middle ear. AOM is predominantly caused by *Streptococcus pneumoniae*, *Heamophilus influenza* and *Moraxella catarrhalis* (Qureishi *et al.*, 2014). However, *Pseudomonas aeruginosa* and *Staphyloccocus aureus* are the most common aerobic microbial isolates in patients with CSOM, followed by *Proteus vulgaris* and *Klebsiella pneumoniae* (Sattar *et al.*, 2012; Aduda *et al.*, 2013; Prakash *et al.*, 2013).

Otitis media when not treated properly or paid attention to can lead to several conditions ranging from mild to fatal. Most severe complications are infratemporal (such as mastoiditis) while others are intracranial (mainly intracranial abscess and meningitis) which ultimately may lead to brain damage or death. Due to the alarming increase in the incidence of antibiotic-resistant microorganisms as a result of the incessant use or abuse of antibiotics, there is a need for an alternative therapy to antibiotics which is cost effective and will help reduce exposure to high dosage levels of antibiotics so as to minimize side effects. The increase in the emergence of antibiotic-resistant pathogens (Westh *et al.*, 2004) has called for the need to carry out research on safer phytomedicines and biologically active compounds with acceptable therapeutic index for the development of novel drugs. Traditional uses of plants have led to investigating their bioactive compounds, which have resulted in the detection of a significant number of therapeutic properties (Sharma *et al.*, 2010).

Essential oils (EOs) which are complex mixtures of volatile compounds produced by plants are characterized by a strong odor and are formed as secondary metabolites (Bakkali *et al.*, 2008). Tiwari *et al.*, (2009) opined that plants produce an array of secondary metabolites that can be found in the edible, medicinal, and herbal plants and their derived essential oils (EOs). These secondary metabolites possess various benefits including antimicrobial properties against pathogenic and spoilage microbes. EOs and other plant extracts have been screened as potential sources of new antimicrobial compounds, alternatively to current antibiotics/disinfectants; or as agents used to promote food conservation (Kon and Rai, 2012; Rios and Recio, 2005; Seow *et al.*, 2014).

Cloves (*Syzygium aromaticum*, syn. *Eugenia aromaticum* or *Eugenia caryophyllata*) are the aromatic dried flower buds of a tree in the family *Myrtaceae* (Chaieb *et al.*, 2007). It is commonly known as *Konafuru* in Yoruba language and is very common in the Northern part of Nigeria, West Africa. Cloves are used as a carminative, to increase hydrochloric acid in the stomach and to improve peristalsis (Phyllis and James, 2000). It is also used in dentistry where the essential oil of clove is used as anadyne for dental emergencies (Prashar *et al.*, 2006). Clove buds and their essential oils have been associated with antimicrobial and antioxidant properties (Fu *et al.*, 2007). The essential oil extracted from the dried flower buds of cloves is used for acne, warts, scars and parasites. Research has shown that clove oil is an effective mosquito repellent (Trongtokit *et al.*, 2005). The clove oil is also used as a topical application to relieve pain and to promote healing and also finds use in the fragrance and flavouring industries (Chaieb *et al.*, 2007a). However, clove oil is toxic to human cells (Prashar *et al.*, 2006). Saeed and Tariq (2008) reported that “the lethal oral dose is 3.752 g/Kg body weight” and if ingested or injected in sufficient quantity, it can cause life threatening complications, including Acute Respiratory Distress Syndrome, Fulminant Hepatic Failure and Central Nervous System disorder. The strong antioxidant activity of Clove and Eugenol can be comparable to the activities of the Butylated hydroxyl anisole (BHA) and Pyrogallol (Dorman and Dean, 2000).

Drug-discovery research has incorporated a wealth of molecular modeling methods via the integration of computational and experimental strategies for the identification and development of novel therapeutic compounds (Leonardo *et al.*, 2015). *In silico* studies are useful in predicting the orientation and binding affinity of ligands at the active site of a receptor target (Lopez-Vallejo *et al.*, 2011).Hence the aim of this study is to determine the antibacterial effects of the essential oil of *Syzgium aromaticum* on both Gram positive and Gram negative multi-drug resistant bacteria isolated from children with otitis media using *in silico* and *in vitro* approach.

## Materials and Methods

### Ethical Approval

Ethical approval was obtained at the Health Research and Ethics Committee of Ogun State Hospital, Ijaiye, Abeokuta, Nigeria, with approval number: SHA/RES/VOI.2/167 on the 6^th^ of April, 2017. Parental consent was also obtained from parents of each patient whose sample was collected.

### Collection of samples

Ear swab samples were obtained from 120 children between the age range of 0-5 attending Ogun State Hospital, Ijaiye, Abeokuta, Nigeria and having presented with otitis media. The ear swab samples were immediately transported using buffered peptone water as transport medium to the laboratory for further isolation. Dried flower buds of *Syzgium aromaticum* were purchased and prepared for extraction.

### Extraction of oils from the plants

Essential oils of clove were obtained by hydrodistillation using a vertical hydrodistillation unit (Amel *et al.*, 2015). This was done using the Clavenger’s apparatus (which comprises of the heating mantle, round bottom flask (3000mls), condenser and the separating funnel). During hydrodistillation, the clove buds were heated to boil in the round bottom flask at temperature of 50°C. The essential oils were trapped in the condenser, pipette and dried over anhydrous sodium sulphate and stored in an amber bottle in the refrigerator at 4°C.

### Isolation and Identification of Bacteria

The characterization and identification of isolates was based on macroscopic morphology, microscopic morphology and several biochemical tests.

### Antibacterial Assay

### Antibiotic Susceptibility testing using disc diffusion method

Susceptibility to antibiotics was assessed using the Kirby-Bauer disc diffusion technique. The results were interpreted according to Clinical and Laboratory Standards Institute (CLSI) (CLSI, 2010). The panel of antibiotics used include: (Tetracycline, Amikacin, Gentamycin, Nalidixic acid, Amoxicillin/Clavulanic acid, Trimethoprime/Sulfomethoxazole, Ciprofloxacin, Chloramphenicol, Ampicillin, Cefoxitin). The suspension of the test organism in nutrient broth was matched with 0.5 McFarland turbidity standards to give a concentration of 1.0 x 10^8^ CFU/ml; while the inoculated plates were incubated at 35°C for 18 – 24 hours. The degree of susceptibility of the bacterial isolates to each antibiotic was determined.

### Sensitivity testing of the essential oils on the bacterial isolates

The susceptibility of microorganisms to the essential oils of *Syzgium aromaticum* was determined using agar well diffusion method as described by Gupta *et al.*, (2008) and Ajayi *et al.*, (2008). This was done in duplicates. The zone of inhibition on each plate was examined and measured in millimeters.

### Determination of Minimum Inhibitory Concentrations (MIC) and Minimum Bactericidal Concentrations of the Essential oils on bacterial isolates

The method used to determine the inhibitory activity of clove essential oil was similar to that as described by Adukwu *et al* (2012) and Akinpelu and Onakoya, (2006) with a few modifications. This assay was done to determine the lowest concentration of the oil that will inhibit microbial growth. Two-fold dilutions of each essential oil was prepared to give concentrations range of 0.003% - 100% (v/v) with methanol as solvent. After which 2ml of the different concentrations was added to 18ml pre-sterilized molten nutrient agar. The molten nutrient agar was poured into sterile Petri dishes and allowed to set. The surface of the media was allowed to dry before streaking with the 18 h old standardized bacteria culture. The plates were later incubated at 37°C for 72 h after which they were examined for the presence or absence of growth. The MIC value was taken as the lowest concentration that prevented the growth of the bacteria.

From the result of the MIC, plates that showed no growth were sub-cultured onto sterile nutrient agar plates and incubated for 48 hours for bactericidal activity. The least concentration that did not show any growth on the incubated nutrient agar plates is the MBC.

### Determination of Rate of Kill

The killing rate of clove essential oil on *Klebsiella pneumoniae* and *Staphylococccus aureus* was done using the protocol as described by Odenholt *et al.*, (2001) and Obuotor *et al.*, (2021).

### Determination of Nucleotide Leakage from test organisms by the essential oil of *Syzgium aromaticum*

The method as described by Akinpelu *et al.*, 2016 was used to determine the leakage of the nucleotides from the test cells. Cells of *Staphylococcus aureus* and *Klebsiella pneumoniae* from 18-hour old nutrient broth culture were separately washed with 0.9% (w/v) normal saline. Standardized washed suspension of the test organisms (inoculum size approximately 10^8^ cells) were treated with various concentrations of the fractions and placed in the centrifuge at 10,000 rpm for 10 minutes. The optical density of the supernatant was thereafter detected at 260 nm wavelength using a spectrophotometer. Standardized organism treated with methanol (solvent) only was used as control.

### Determination of Protein Leakage from the test organism by the essential oil of *Syzgium aromaticum*

Cells of *Klebsiella pneumoniae* and *Staphylococcus aureus* that have been incubated for 24hours in nutrient broth were separately washed in 0.9%w/v normal saline. The washed suspension of bacterial cells (inoculums size approximately 10^8^ cells 0.5 McFarland standards) was treated with various concentrations of the fraction relative to MICs at various time intervals of 90 minutes. Each suspension was then centrifuged at 7000rpm and supernatant collected was assayed for protein using Bradford (1976) method. In assaying for protein, 0.4mL Bradford reagent was added to 1.6mL sample (0.2mL supernatant plus 1.4mL sterile distilled water) to make up 2mL total volume. Optical Density (OD) of the resulting solution was thereafter taken at 595nm after 5mins. The OD of each of the samples calculated from the equation of the best linear regression line obtained from the graph of bovine serum albumin (BSA) standard curve. Standardized organism treated with methanol (the solvent) only was used as control.

### *In vitro* evaluation of the clove EO and ciprofloxacin

The effects of the essential oil of *Syzgium aromaticum* in combination with ciprofloxacin against *Staphylococcus aureus* and *Klebsiella pneumoniae* were evaluated using the “Overlay Inoculum Susceptibility Disc method” as described by Okore, (2010) with modifications. Pre – sterilized nutrient agar was mixed with the MIC x 1 value of the essential oil of *Syzgium aromaticum* and poured into sterile clean Petri dish as the base agar and allowed to solidify after which about 2 ml of molten nutrient agar was inoculated with the test organism and shaken gently to ensure uniformity of the cells in the medium. The inoculated medium was then poured on the surface of the base agar to form a thin uniform layer, the overlay inoculum agar, the antibiotic disc (ciprofloxacin (oxoid)) was placed on the agar medium at the center of the solidified agar plates. Two control plates were prepared. Control A contained sterile nutrient agar with the overlay inoculum agar and the antibiotic disc, while control B contained the base agar (without antibiotics) and the overlay inoculum. The three plates in the set were left at room temperature for 1 h to allow for pre-diffusion, and then incubated at 37°C for 24 h. The zone of inhibition formed on the test plates was used to determine the combined effect of the two antibiotics, by comparing it with the result in control B. Synergism is obtained when the diameter of the zone of inhibition in the test plate is greater than that in + control (Ciprofloxacin) by at least 19%; lower than 19% indicates additivity, equal to control B indicates indifference; when it is less than control B, there is antagonism.

% increase was calculated using the formula;


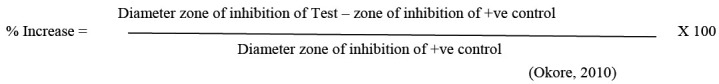


### Phytochemical screening of the essential oils

Clove essential oil was subjected to phytochemical screening using the method as described by Kalaivani and Vidhya, (2014). This method was used to test for saponins, tannins, terpenoids, glycosides, alkaloids, flavonoids and reducing sugars.

### Antioxidant activity of the essential oils

### Radical Scavenging ability

The “radical scavenging” ability of the oil was determined using the stable radical DPPH (2,2-diphenyl-1-picrylhydrazyl hydrate) as described by Brand-Williams *et al.*, (1995). The reaction of DPPH with an antioxidant compound which can donate hydrogen, leads to its reduction (Blois, 1958). The change in colour from deep violet to light yellow was measured using a spectrophotometer at 517nm.

### Determination of Total Antioxidant Capacity

This method is based on the reduction of Molybdenum (VI) to Molybdenum (V) by the extract and the subsequent formation of a green phosphate/Molybdenum (V) complex at an acidic pH (Prieto *et al.*, 1999).

### Determination of Ferric Reducing Antioxidant Power (FRAP)

The FRAP assay was carried out according to the method described by Benzie and Strain (1999) which is based on “the reduction of ferric – tripyridyltriazine complex to its blue ferrous coloured form” due to electron transfer in the presence of antioxidant.

### Metabolite profiling with GCMS Analysis

The Physicochemical properties of *Syzgium aromaticum* was identified using “Gas Chromatography Mass Spectrometry (GC-MS) analyzer (Shimadzu GC-MS-QP 2010 Ultra)”. SLB-5ms Column fused with silica capillary 0.20mm X 30.0m with film thickness 0.20μm was used for this purpose. The oil was introduced into the equipment and varying components of the oil with their chemical properties were detected at different peaks.

### Drug Metabolism and Pharmacokinetics

### ADMET studies

The ADMET (absorption, distribution, metabolism, elimination, and toxicity) studies of compounds obtained from GC – MS analysis were carried out using “pkCSM tool (http://biosig.unimelb.edu.au/pkcsm/prediction)” and Swiss ADME (Daina *et al*. 2017; Pires *et al*. 2015). Evaluation of their physicochemical properties was first done to decide the Pharmaceutical Active Ingredients (PAIs) utilizing the “Lipinski rule of five” (Molecular weight, logarithms of partial coefficient, hydrogen bond donor (HBD) and hydrogen bond acceptor (HBA)) (Lipinski et al. 1997). Thereafter, the “canonical SMILES” for the molecular structure were acquired from “PubChem” (https://bar chem.ncbi.nlm.nih.gov). The results obtained from this were screened for pharmacokinetic properties.

### Ligand preparation

The SDF designs of the ligands were recovered from the “PubChem information base (www.pubchem.ncbi.nlm.nih.gov)” (Kim *et al*. 2019). The ligands were converted to .pdb format using the “PYMOL atomic illustrations framework (1.7.4.5 Edu)” (DeLano 2002).The ligand atoms were thereafter completely converted to the dockable .pdbqt format using the “Autodock vina program”

### Enzyme preparation

The crystal structure of the enzymes of the test organisms (7BYE of *Klebsiella pneumoniae*, 4HBL of *Staphylococcus* *epidermidis*, 3PR7 of *Moraxella cattarhalis*, 4MCX of *Proteus vulgaris*. 6SPD of *Pseudomonas aeruginosa*, 6H2L of *Proteus mirabilis* and 2NOJ of *Staphylococcus aureus)* were downloaded from the “protein data bank (www.rcsb.org)”. The crystals were prepared using the method described by Berman *et al.*, (2000) and the enzyme was subsequently saved into .pdbqt format in preparation for molecular docking.

### Molecular docking

The protocol of Trott and Olson (2010) was employed in the molecular docking of the ligands with the enzymes from the test organisms. The free binding (∆G bind) was calculated using the protocol as reported by Isa *et al*. (2020). The binding energies of each compounds were identified and further graphical analysis was obtained using “Discovery Studio Visualizer, BIOVIA, 2016”.

## Results

### Prevalence of organisms implicated in Otitis Media

**Figure 1:**
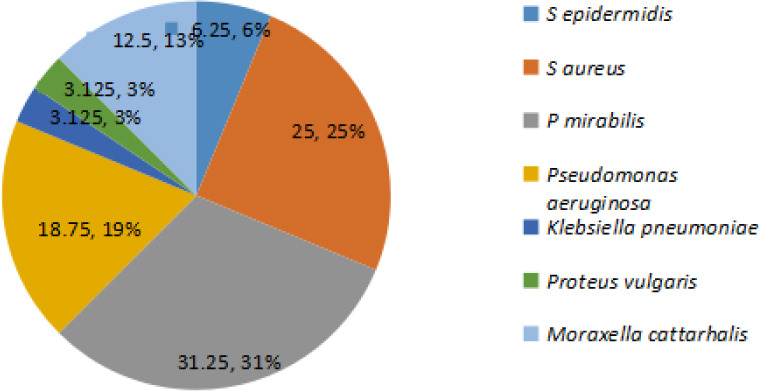
Frequency of isolated organisms from ear swab samples

The prevalence of the organisms isolated from Otitis media ear swab samples as shown in Figure 1 shows that *Proteus mirabilis* had the highest occurrence of 31.25% while *Proteus vulgaris* and *Klebsiella pneumonia* had the lowest occurrence of 3.13%. *Staphyloccoccus aureus, Pseudomonas aeruginosa*, *Moraxella catarrhalis* and *Staphylococcus epidermidis* had occurrences of 25%, 18.75%, 12.5% and 6.25% respectively.

### Characteristics of the Essential oils obtained from *Syzgium* aromaticum

The oil obtained from *Syzgium aromaticum* was a colourless, dense liquid with a sharp odour and a percentage yield of 4%.

### Sensitivity testing of antibiotic disc on bacterial isolates

The antibiotic sensitivity testing on bacterial isolates results are shown in Table 1. It was observed that *Pseudomonas aeruginosa* and *Moraxella cattarhalis* were both resistant to 7 out of the 10 antibiotics used, followed by *Klebsiella pneumoniae* and *Proteus vulgaris* which were resistant to 6 antibiotics. *Staphylococcus aureus* and *Proteus mirabilis* were susceptible to 6 antibiotics in contrast to the other bacterial isolates.

**Table 1:** Antibiotic disc sensitivity testing on bacterial isolates (in mm)

Isolates	FOX (mm)	CN (mm)	CIP (mm)	AMC (mm)	SXT (mm)	NA (mm)	AMP (mm)	C (mm)	AMK (mm)	TET (mm)
***Klesiella pneumonia***	12±1.4	23.5±0.7	26±0	15.5±0.7	22±1.4	21.5±0.7	11.5±2.1	6±0	21.5±0.7	6±0
***Staphylococcus epidermidis***	26±0	30±0	12.5±0.7	33±2.8	7.5±2.1	6±0	32.5±0.7	31±1.4	23±0	13±0
***Pseudomonas aeruginosa***	6±0	23.5±0.7	28±1.4	6±0	6±0	11.5±0.7	6±0	14±0	21±1.4	11.5±0.7
***Proteus vulgaris***	24±0	24±0	18.5±2.1	8±0	6±0	22.5±0.7	6±0	7±0	23±0	6±0
***Proteus mirabilis***	25±0	6±0	18±0	27.5±0.7	17±1.4	20.5±3.5	19.5±0.7	18.5±0.7	6±0	7±0
***Moraxella catarrhalis***	7±0	8±1.4	31±1.4	35±1.4	6±0	6±0	25±0.7	13.5±2.1	11.5±0.7	19.5±0.7
***Staphylococcus aureus***	28.5±2.1	28.5±0.7	25.5±0.7	35.5±0.7	6±0	12.5±0.7	30.5±0.7	19.5±2.1	28±0	27.5±0.7

Key: FOX- Cefoxitin (30μg); CN- Gentamycin (120μg); CIP- Ciprofloxacin (5μg); AMC- Amoxicillin/clavulanic acid (30μg); NA- Nalidixic acid (30μg); AMP- Ampicillin (10μg); C- Chloramphenicol (30μg); AMK- Amikacin (30μg); TET- Tetracycline (30μg); SXT- Sulfomethoxazole/Trimethoprin (25μg).

### Antibacterial activities of the essential oil of *Syzgium aromaticum* against bacterial isolates.

**Figure 2:**
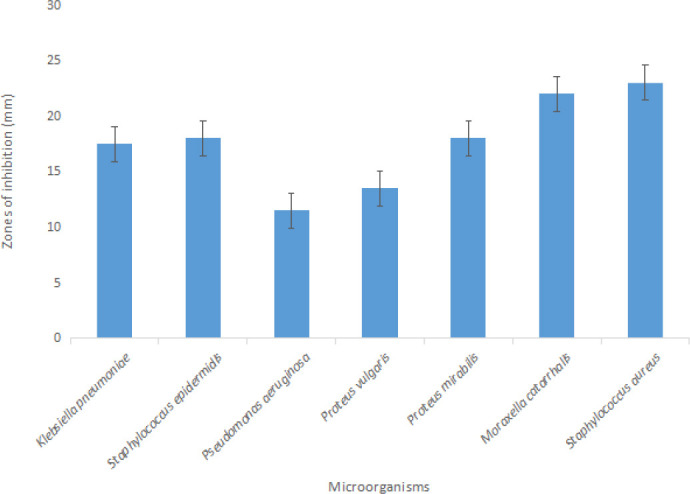
Antibacterial activities exhibited by the essential oil of Clove.

The antibacterial activities of the essential oil of *Syzgium aromaticum*, against bacteria isolated in this study, is as shown in Figure 2. All of the bacterial isolates were susceptible to the essential oil of *Syzgium aromaticum* with *Staphylococcus aureus* having the highest zone of inhibition of 23.0 ± 2.8 and *Pseudomonas aeruginosa* having the lowest zone of inhibition of 11.5 ± 0.7.

### Minimum Inhibitory Concentration (MIC) and Minimum Bactericidal Concentration (MBC) of *Syzgium aromaticum* essential oil against test isolates.

The Minimum Inhibitory Concentration and Minimum Bactericidal Concentration exhibited by the essential oil against the bacterial isolates are as shown in Table 2. The MIC exhibited by the essential oil of *Syzgium aromaticum* ranged between 0.78%v/v and 3.13%v/v while MBC ranged 0.78%v/v and 1.56%v/v.

**Table 2:** The Minimum Inhibitory Concentrations and Minimum Bactericidal Concentrations exhibited by the essential oils of Clove.

S/N	Isolates	MIC (%v/v)	MBC (%v/v)
1	*Klebsiella pneumoniae*	**0.78**	**0.78**
2	*Staphylococcus epidermidis*	**1.56**	**3.13**
3	*Pseudomonas aeruginosa*	**1.56**	**1.56**
4	*Proteus vulgaris*	**0.78**	**0.78**
5	*Proteus mirabilis*	**1.56**	**3.13**
6	*Moraxella catarrhalis*	**3.13**	**3.13**
7	*Staphylococcus aureus*	**1.56**	**1.56**

### Killing rate of the essential oil of *Syzgium aromaticum* on *Staphylococcus aureus* and *Klebsiella pneumoniae*

**Figure 3:**
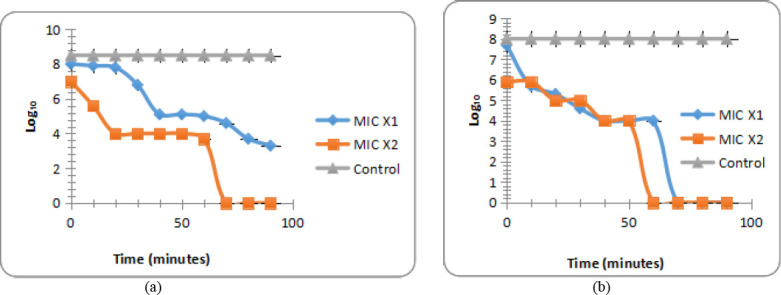
Time kill kinetics of clove essential oil against *Staphylococcus aureus* (Fig 3a) and *Klebsiella pneumoniae* (Fig 3b).

The killing rate exhibited by the essential oil of *Syzgium aromaticum* at 1 x MIC and 2 x MIC as shown in Figure 3 shows that as the time of exposure increases, there is an incessant decrease of cell population. For *Staphylococcus aureus* (Figure 3a) at MIC x 1, the colony forming units (cfu) counted at 0 minute was 1 x 10^8^ cfu/ml which then decreases progressively to 2 x 10^3^cfu/ml at 90 minutes. Also, at MIC x 2, 9.3 x 10^6^cfu/ml was counted at 0 minutes and at 70 minutes, there were no colony forming units observed. In addition, for *Klebsiella pneumoniae* (Figure 3b) at MIC x 1, the colony forming units counted at 0 minutes was 4.5 x 10^7^cfu/ml which also progressively decreases to no colony forming units observed at the 70-minute time exposure. Similarly, at MIC x 2 a total of 8.4 x 10^5^cfu/ml was counted at 0 minute following which a rapid decrease leading to no colony forming units was observed at the 60 minute time exposure.

### Mechanism of action of the essential oil of *Syzgium aromaticum*

**Figure 4:**
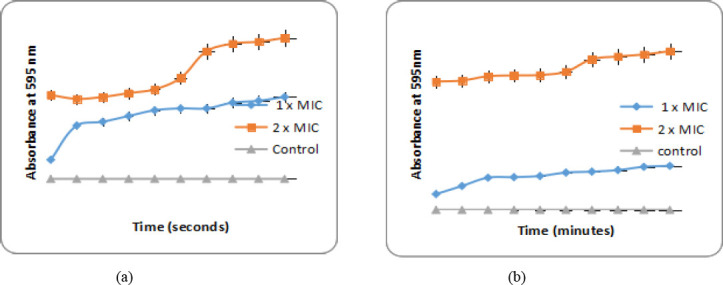
Nucleotide leakage of cells of *Staphylococcus aureus* (a) and *Klebsiella pneumonia* (b).

Nucleotide leakage as a function of mode of action indicates that the rate of leakage increases as the exposure increases. An increase in the absorbance values of *Staphylococcus aureus at* MIC x 1 and MIC x 2 is shown in Figure 4a meanwhile the control did not exhibit any significant change in the absorbance values. Figure 4b also shows a rise in the absorbance values of *Klebsiella pneumoniae* at MIC x 1 and MIC x 2.

**Figure 5:**
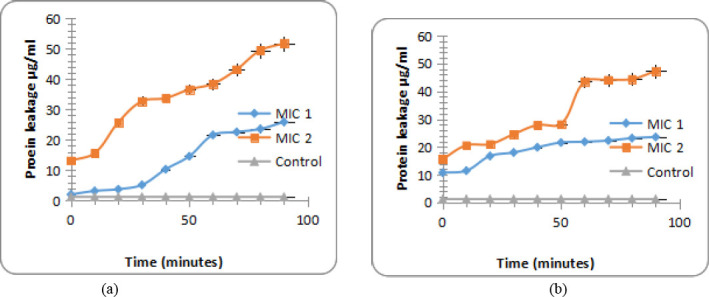
Rate of protein leakage from *Staphylococcus aureus* (a) and *Klebsiella pneumonia* (b)

Furthermore, the protein leakage per time interval in *Staphylococcus aureus* as shown in Figure 5a depicts a steady increase in protein leakage concentration as the time of exposure increases. For MIC x 1 at 0 minute, 10.7μg/ml was recorded and this increased to 23.5μg/ml at 90 minutes while for MIC x 2 there was an increase from 15.6μg/ml at 0 minute to 47.2μg/ml at 90 minutes. Also for *Klebsiella pneumoniae* as shown in Figure 5b, at 0 minute for MIC x 1 and MIC x 2 the values of 2μg/ml and 13.4μg/ml were recorded respectively while at 90 minutes for MIC x 1 and MIC x 2 the values of 25.8μg/ml and 51.7μg/ml were recorded respectively. Controls for both organisms did not show any significant change in concentration.

### The effect of the combination of the essential oil of *Syzgium aromaticum* with ciprofloxacin on bacteria isolates.

The impact of the combination of the essential oil of *Syzgium aromaticum* with the commercial antibiotic Ciprofloxacin (Oxoid) is shown in Table 3.

**Table 3:** The effect of the combination of the essential oil of *Syzgium aromaticum* with ciprofloxacin on bacteria isolates.

Diameter zone of inhibition (mm)			
Organisms	Test	Ciprofloxacin	%increase	Effect
*Staphylococcus aureus*	31	32	-	Antagonism
*Proteus vulgaris*	28	18	55.6%	Synergism
*Proteus mirabilis*	22	20	10%	Additivity
*Pseudomonas aeruginosa*	33	39	-	Antagonism
*Klebsiella pneumonia*	58	41	41.5%	Synergism
*Moraxella catarrhalis*	21	21	-	Indifference
*Staphylococcus aureus*	30	27	11.1%	Additivity

### Antioxidant activity of the essential oil of *Syzgium aromaticum*.

Table 4 shows the antioxidant capacity of clove essential oil. The essential oil of *Syzgium aromaticum* had TAC value of 0.12mg/ml. However, reverse is the case in the Ferric Reducing Antioxidant Power (FRAP) values as the essential oil of *Syzgium aromaticum* had a higher value of 1.83mg/ml For Di-phenyl Picryl Hydrazyl Hydrate (DPPH) assay, the 1.13mg/ml was recorded for *Syzgium aromaticum* essential oil.

**Table 4:** Antioxidant assay of essential oils

S/N	ANTIOXIDANT ASSAY	*Syzgium aromaticum* oil (mg/ml)
1.	Di-phenyl Picryl Hydrazyl Hydrate (DPPH) (mg/ml)	1.13±0.65

2.	Ferric Reducing Antioxidant Power (FRAP) (mgAAE/ml)	1.83±1.05

3.	Total Antioxidant Capacity (TAC) (mg/ml)	0.12±0.07

### Phytochemical screening of the essential oils

The phytochemical screening of the essential oil of *Syzgium aromaticum*, indicated the presence of several phytochemical compounds in the oils including; Flavonoids, Sterols, Phenols, Carbohydrates and Alkaloids. Table 5 shows the phytochemical screening results for the essential oils.

**Table 5:** Phytochemical screening of the essential oils

Phytochemicals	Tannin	Saponin	Terpenoids	Flavonoids	Glycosides	Sterols	Phlobatannns	Phenols	Carbohydrates	Alkaloids	Resins
**Clove Essential oil**	-	-	-	+	-	+++	-	++	+	+	-

Key: (-) Negative test; (+) Weak positive test; (++) Positive test; (+++) Strongly positive test.

### Physicochemical analysis of the Essential oils

**Figure 6:**
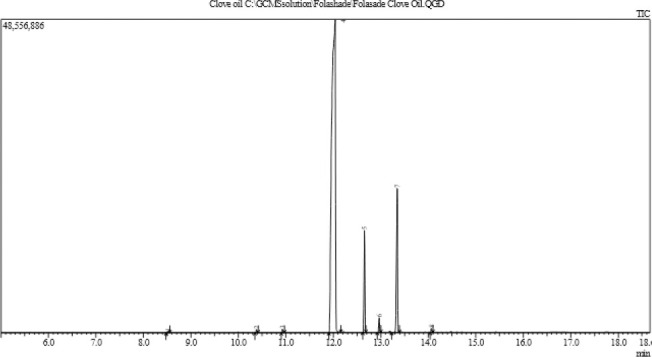
Graph showing peaks of components of *Syzgium aromaticum* essential oil.

The result from Gas Chromatography-Mass Spectroscopy (GC-MS) analysis of the essential oil of *Syzgium aromaticum*, is as shown below. It can be depicted that clove essential oil has a total of 8 compounds which are represented as peaks in Figure 6.

**Figure 7:**
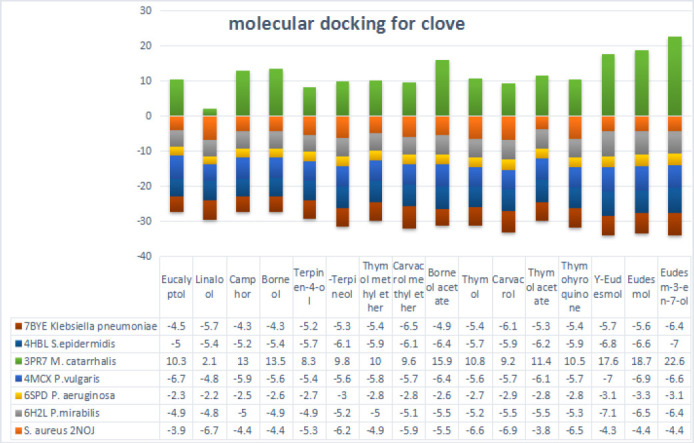
Molecular docking results of the various proteins of the bacteria isolated from samples and their corresponding performances when docked with compounds identified from CEO (after they passed the ADMET test and synthetic accessibilities verified)

**Figure 8:**
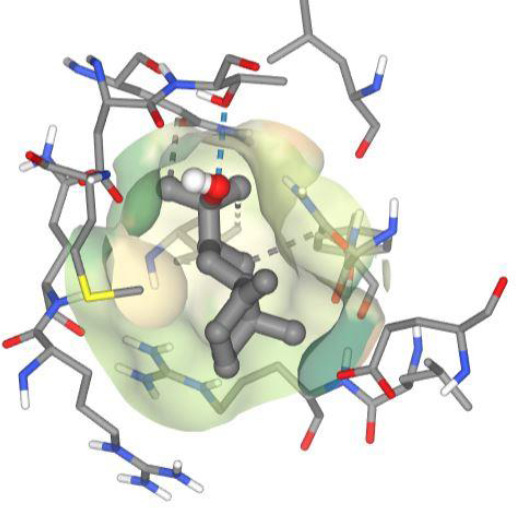
Image showing the interaction of eudesmol and the 4MCX of *P. vulgaris*.

**Figure 9:**
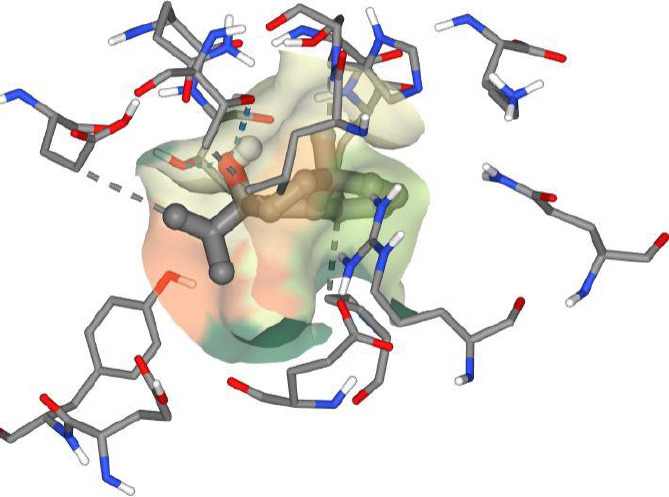
Image of the interaction of Eudesm-3-en-7-ol and the 4HBL of *S. epidermidis*.

**Figure 10:**
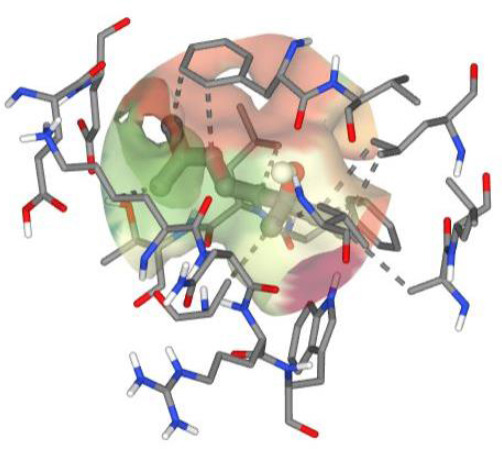
Image showing the interaction of Linalool and 7BYE of *K.Pneumoniae*

## Discussion

A total of 7 bacterial species were isolated and it was observed that *Proteus mirabilis* indicated the highest percentage of occurrence after which *Staphylococcus aureus* and *Pseudomonas aeruginosa* which is similar to the findings of Ilechukwu *et al.*, (2014), Ako-Nai *et al.*, (2002) and Oni *et al.*, (2001). However, these findings are in contrast with the trend in the developed world where non-typeable *Heamophilus influenza*, *Streptococcus pyogenes* and *Moraxella catarrhalis* assume important predominant roles in Otitis media (Casey *et al.*, 2004).

Clove essential oil (CEO) showed inhibitory activity against all bacteria however moderate activity was observed in some. Similar antibacterial pattern of CEO against *S. aureus* was also reported in a study done by Abdullah *et al.*, (2015). Thosar *et al.*, (2013) also reported strong inhibitory effects of clove EO at low concentrations against all organisms tested as compared with other oils such as lavender and peppermint oil.

Haripriyan *et al.*, (2018), studied the effect of clove bud oil on “four *Pseudomonal* proteases – elastase A, elastase B, Protease IV and alkaline protease” – each known to play a key role in *Pseudomonal* disease pathogenesis and it was established that clove bud oil exhibits “an immune-boosting property which supports its anti-virulence capacity and thus provides a two-pronged approach by which it inhibits *P. aeruginosa* infections”. Eugenol a major component of CEO has been proven effective in combating several pathogens such as *S. typhyi*, *P. mirabilis* (Devi *et al.*, 2010; Devi *et al.*, 2013)*, E. coli*, *S. aureus* *P. aeuriginosa* (Walsh *et al.*, 2003) by altering the integrity of their cell membranes (Devi *et al.*, 2013), but according to the toxicity test conducted, it is not a suitable druggable compound. Hence, compounds from CEO such as linalool, cavacrol, eucalyptol, thymol, eudesmol and its gamma counterpart should be synthesized and clinically validated as potential new antimicrobial agents. Especially with regards to pediatric otitis media.

“The ability of plant extract to kill or eliminate microorganisms at the shortest period of time” is generally accepted definition of bactericidal activity in antibiotics (Pankey and Sabath, 2004). The results of Time kill kinetic studies of Clove EO against *S. aureus* and *K. pneumoniae* showed a steady decrease in cell population for both bacteria species with a continuous increase in time at intervals of 10 minutes. A 100% kill for *Staphylococcus aureus* was exhibited by CEO at a concentration of MIC x 2 at 70 min contact time while 100% kill of *Klebsiella pneumoniae* at a concentration of MIC x 1 within the shortest time of 70 min contact time was observed. The hydroxyl group OH of eugenol present in CEO contributes to its inhibitory effect as it binds to and affects the properties of proteins, thus inhibits the activity of some enzymes such as ATPases which may be important for cell kill at high eugenol concentrations because energy generation needed for cell recovery is impaired (Gill and Holley, 2006a).

Nucleotide leakage was observed in the cell wall of *S. aureus* and *K. pneumoniae*. This is indicating that there is a loss in purine and pyrimidine bases through a damaged cytoplasmic membrane (Stojkovic *et al.*, 2013). When the test isolates were treated with clove essential oil, there was a continuous increase in rate of leakage as the exposure time increased. This is an indication of monophasic effects as described by Akinpelu *et al*. (2016). The nucleotides have strong UV absorption at 260 nm, membrane integrity can be determined through the detection of absorbance at this wavelength (Stojkovic *et al.*, 2013). A steady increase in the leakage of protein content in the bacteria cells with increase in time exposure was also observed in this study. A study performed by Oyedemi *et* al., (2009) on a wide group of bacteria revealed that eugenol (a major component of clove EO) caused cell lysis by damaging the cell wall and membrane which led to the leakage of protein and lipid contents after 120 min of time exposure. Gill and Holley (2006b) also corroborated this findings.

CEO was observed to consist of 8 major compounds Eugenol (80.98%), Phenol, 2-methoxy-4-(2-propenyl)-, acetate (11.52%), Caryophyllene (6.00%), Humulene (0.81%), Caryophyllene oxide (0.22%), Phenol, 4-(2-propenyl) (0.22%), Methyl salicylate (0.16%) and 6-Methyl-2-Heptanol, acetate (0.10%). This is in consonance with the work of Omidbaigi *et al.*, (2007) who reported eugenol, caryophyllene and eugenol acetate as the major components of CEO however while contrasting to the work of Naveed *et al.*, (2013) who reported the presence of oxygenated monoterpenes and eucalyptol as the major component with eugenol in minor quantity. The variations in the chemical composition of the oil might have been due to the existence of different species and also the differences in agro-climatic conditions (Singh *et al.*, 2008; Anwar *et* al., 2009; Singh *et al.*, 2010; Lee, 2016).

Preliminary phytochemical analysis of the essential oils revealed the presence of flavonoids, sterols, phenols, carbohydrates and alkaloids in CEO. This was also reported by Dahiya and Soni, (2014), Ahmed, (2016) and Hemalatha *et al.*, (2016). In contrast to this study, Oviya *et al.*, (2016) reported the presence of tannin and glycosides. Phenols present in high contents have been found to denature proteins and react with cell membrane phospholipids changing their permeability (Briozzo *et al.*, 1989). Peter and Wong (2006) opined that phytoconstituents obtained in the essential oil could account for its antioxidant and antimicrobial activity. Variations in phytochemical properties may be attributed to geographical conditions, climate, harvest season, period of distillation and extraction method used.

* In silico* analysis conducted in this study revealed that the components of the essential oils are potential lead molecules in the inhibition of bacterial growth and thus justify its use in traditional folklore medicine. It was observed that of the 36 phytocompounds identified by the GC-MS, only 16 of them passed the ADMET test. Also, out of these 16 compounds, their binding energy varies for each organism. In more specific terms: Linalool and cavacrol had best performance with *S*. *aureus, g* amma-eudesmol and eudesmol were top performer for *P. mirabilis, S. epidermidis* and *P. aeruginosa*. Eucalyptol, gamma-eudesmol and eudesmol for *P. vulgaris*. Finally, for *Klebsiella pneumoniae* Eudesm-3-en-7-ol and cavacrol methyl ether were the top performing phytocompounds. These compounds had the lowest binding energies, hence their potential to inhibit the synthesis of important proteins required by bacteria for survival. Their synthetic accessibilities were also put into consideration alongside the ADMET analysis. The ability of these phytocompounds (ligands) to bind to the active sites of enzymes, inhibits their substrates from binding, thereby interrupting the synthesis process of the proteins that play a key role in the survival or resistance mechanism of the bacteria. The lower the binding energy as compared to the binding energy of the normal substrate of an enzyme, the higher potential the phytocompound (ligand) possesses to interfere with synthesis.

## Conclusion

Clove essential oil has long been known for its antibacterial potency and this study further confirms it. *In silico* study is a fast rising antimicrobial method that is proving to create more research options in the quest for alternatives to antibiotic therapy. Several advantages are associated with the use of spices as dietary supplement or alternative medicine including a reduction in the occurrences of antibiotic-resistant bacteria that results from the frequent use of antibiotics (misuse or abuse), a decrease in the cost of treatment and also minimal development of adverse drug reactions. This study reveals that the antibacterial potency of phytocompounds in essential oil is not dependent on its quantity therefore, all components of the oil should be treated and considered as potent until proven not to be. Further independent studies should be carried out on the phytocompounds with high binding energy identified from clove oil as alternative therapy in the treatment of Otitis media.

### Conflict of Interest

The authors declare that there is no conflict of interest associated with this study.

**List of Abbreviations:**
OM –: Otitis Media;
EO –: Essential oil;
Eos –: Essential oils;
MIC –: Minimum Inhibitory Concentration;
MBC –: Minimum Bactericidal concentration.
